# Lumbar spine epidural abscess and facet joint septic arthritis due to *Streptococcus agalactiae*: a case report

**DOI:** 10.1186/s12893-018-0350-2

**Published:** 2018-03-13

**Authors:** Costansia Bureta, Hiroyuki Tominaga, Takuya Yamamoto, Takao Setoguchi, Hideki Kawamura, Satoshi Nagano, Ichiro Kawamura, Masahiko Abematsu, Hironori Kakoi, Yasuhiro Ishidou, Setsuro Komiya

**Affiliations:** 1 Department of Orthopaedic Surgery, Graduate School of Medical and Dental Sciences, Kagoshima University, 8-35-1 Sakuragaoka, Kagoshima, 890-8520 Japan; 2 The Near-Future Locomotor Organ Medicine Creation Course (Kusunoki Kai), Graduate School of Medical and Dental Sciences, Kagoshima University, Kagoshima, Japan; 3 Division of Medical and Environmental Safety, Kagoshima University Medical and Dental Hospital, Kagoshima, Japan; 4 Medical Joint Materials, Graduate School of Medical and Dental Sciences, Kagoshima University, Kagoshima, Japan; 5 Department of Neurosurgery, Muhimbili Orthopaedic and Neurosurgical Institute, P.O. Box 65474, Dar es Salaam, Tanzania

**Keywords:** Spinal epidural abscess, Facet joint septic arthritis, *Streptococcus agalactiae*, Urine retention, Antibiotic administration, Hemilaminectomy

## Abstract

**Background:**

Here we report a rare case of lumbar spine epidural abscess and facet joint septic arthritis caused by *Streptococcus agalactiae*, which had spread to the iliopsoas muscles, leading to urine retention.

**Case presentation:**

A 68-year-old woman with low back pain experienced a sudden onset of bilateral lower limb weakness, it was followed 14 days later by urine retention. At consultation, magnetic resonance imaging and identification of serum β-hemolytic streptococci provided a diagnosis of *Streptococcus agalactiae* infection. She was started on antibiotics. Despite diminishing signs of inflammation, preoperative MRI showed an epidural mass at T12-L4 compressing the cord and involving the paravertebral muscles as well. Group B beta-hemolytic streptococci were detected in both urine and blood. Because of bilateral lower limb weakness and urine retention, T12-L4 hemilaminectomy was performed. The L3/L4 intertransverse ligament resected and abscess drained. Histopathology revealed that inflammatory cells had invaded the facet joint. Group B beta-hemolytic streptococci were identified, confirming the diagnosis. The patient continued with the antibiotics postoperatively, and her health rapidly improved.

**Conclusion:**

Lumbar spine epidural abscess and facet joint septic arthritis caused by *Streptococcus agalactiae* is a clinical emergency, with significant morbidity and mortality especially with delayed diagnosis. A delay in both diagnosis and aggressive treatment can lead to not only severe neurological deficit but also to septicaemia, multiorgan failure, and even death.

## Background

Spinal epidural abscess (SEA) is a spine surgical emergency, with a potential threat of devastating neurologic sequelae, including direct spinal cord compression, vascular compromise, and mechanical spine instability. Despite increased awareness among physicians and magnetic resonance imaging (MRI)-facilitated diagnosis, SEA has rarely been reported to be caused by Group B haemolytic streptococci [1–4]. We report a case of Lumbar SEA with facet joint septic arthritis due to Group B haemolytic streptococcal, that had spread to the iliopsoas muscles, leading to urinary retention. To the best of the authors’ knowledge, this combination has not been reported in the English-language literature.

## Case presentation

A 68-year-old woman with hypertension and a history of a uterine myoma was admitted complaining of severe back pain and pain in both legs for 12 days. She exhibited no lower limb muscle weakness or dysuria.

**Fig. 1:**
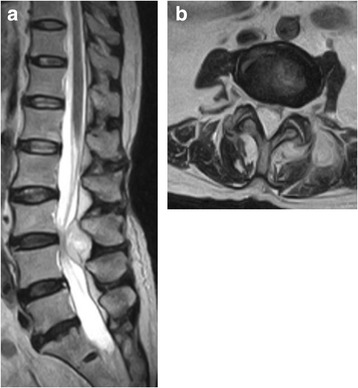
T2-weighted magnetic resonance imaging (MRI) of an abscess in the L3/4 epidural space and paravertebral muscle at the initial diagnosis in our hospital. **a** Sagittal view. **b** Axial view

On physical examination, she was febrile (temperature of 38 °C). There was local tenderness over the lower lumbar region with no signs of skin infection. No lower limb muscle weakness was observed, and there were no urinary symptoms. Hence, she had a score of 7 on the Japanese Orthopaedic Association scale. MRI revealed a mass in the L3/4 left facet, epidural space, and paravertebral muscles (Fig. 1a, b). The erythrocyte sedimentation rate (ESR) was 40 mm/h, C-reactive protein levels (CRP) was 20.97 mg/dL, leukocytosis was 27,710/μL, with 91% neutrophils. Group B beta-hemolytic streptococci (*Streptococcus agalactiae*) were detected in both blood and urine. With a diagnosis of sepsis due to urinary tract infection. We prescribed ampicillin (2 g × 4/day). We did not consider surgery because neither lower limb muscle weakness nor dysuria was present. The signs of inflammation decreased, and febrile symptoms diminished.

**Fig. 2:**
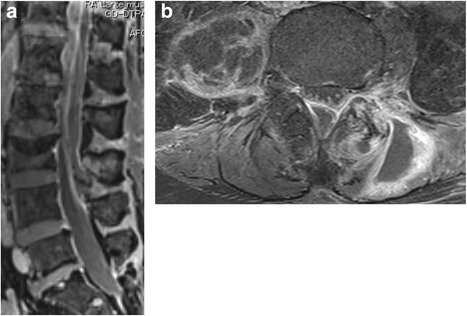
T1-weighted MRI with gadolinium enhancement at the L3/4 level. Two weeks after lumbago onset, the mass (enhanced at the edge) had spread widely to iliopsoas muscles, paraspinal muscles, and the epidural space. **a** Sagittal view. **b** Axial view

One week after starting antibiotic treatment, a gadolinium-enhanced MRI scan of the lower lumbar region revealed an epidural mass (a finding consistent with an epidural abscess), extending from T12 to L4 on the posterior canal and compressing the spinal cord. The mass had also spread to the iliopsoas and paraspinal muscles (Fig. 2a, b). At this time, the patient also exhibited lower limb muscle weakness. The manual muscle test showed that the bilateral iliopsoas, quadriceps, and tibialis anterior muscles were grade 3, and the extensor hallucis longus was grade 2. The patient also exhibited bilateral lateral leg sensory disturbance (4/10) and urinary retention. Her saddle sensory ability remained.

**Fig. 3:**
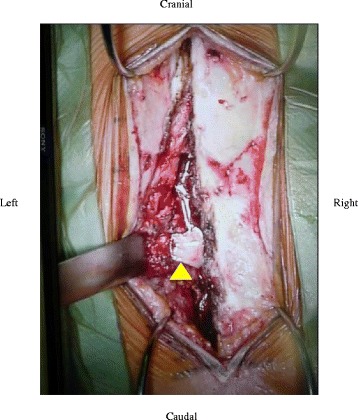
L3/4 facet joint parcel was torn. A white abscess is apparent in the paraspinal muscle. Findings were similar in the iliopsoas muscle and epidural abscess

**Fig. 4:**
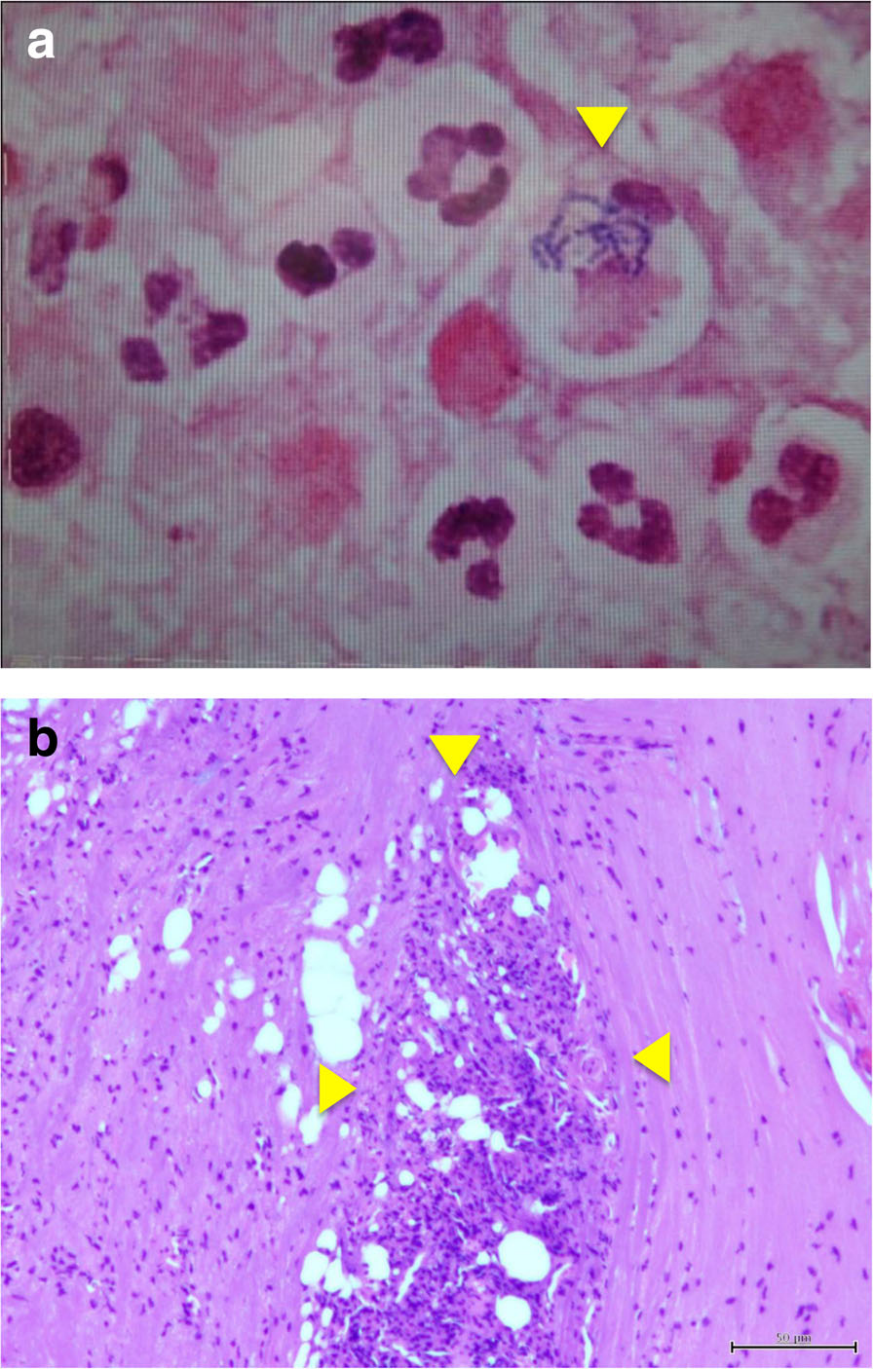
**a** Note the streptococci being engulfed by leukocytes. **b** Pathology examination shows inflammatory cells in bone tissue of the L3/4 facet

Surgery was performed via posterior approach under general anaesthesia. Left-side hemilaminectomy was undertaken from T12 to L4 along with incision of the right intertransverse ligament between L3 and L4. A white abscess was observed in the left L3/4 paraspinal muscles, epidural space between T12 and L4, and the iliopsoas muscles between the right L1-L5 (Fig. 3). The L3/4 facet joint capsule was torn, from which tissue was obtained for pathology examination. Microscopic examination revealed leukocytes in the abscess, and tissue specimen showed streptococcal infection (Fig. 4a, b).

**Fig. 5:**
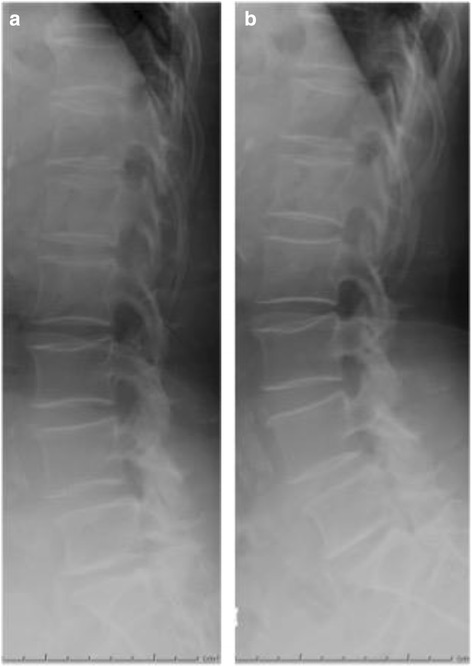
Lateral XP image (**a**) Flexion (**b**) Extension of the lumbar region 15 months after surgery. Note that there was no instability of the lumbar spine

**Fig. 6:**
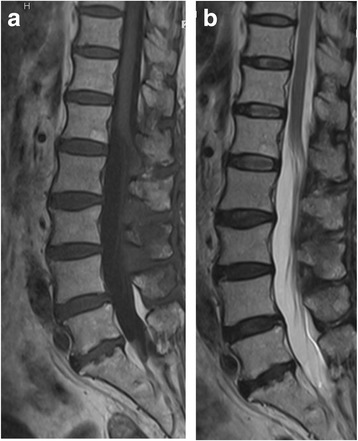
Sagittal view of T1-weighted (**a**) and T2-weighted (**b**) MRI image and a T2- weighted image at the L3/L4 level 47 days after surgery. Note that the abscess has disappeared

Antibiotic administration continued until the abscess disappeared (at 47 days postoperatively). CRP values had dropped to 0.02 mg/dL, and white blood cells count was 5910/μL (normal). Postoperatively, both feet had regained muscular strength (manual muscle test grade 5), and her dysuria disappeared. She did not develop arachnoiditis post-operatively. The Japanese Orthopaedic Association score improved to 27/29 points. Dynamic plain radiographic imaging showed no instability of the lumbar spine 15 months postoperatively (Fig. 5a, b), and MRI showed no evidence of the abscess (Fig. 6a, b).

## Discussion and conclusions

SEAs are uncommon, although the incidence has been increasing (now at 0.2–3/10,000 hospital admissions). SEAs are often associated with intravenous drug abuse, diabetes mellitus, previous spinal procedures or invasive diagnostic/therapeutic procedures, infectious endocarditis, trauma, conditions that could lead to immune suppression (e.g. acquired immunodeficiency syndrome), obesity, chronic steroid use, chronic renal failure, Crohn’s disease, systemic and local infection, malignancy, and alcoholism [1–3, 5, 6] . Our patient had a uterine myoma, which may have caused the urinary tract infection that lead to lumbar SEA with facet joint septic arthritis.

The leading bacterial causes of SEA are *Staphylococcus aureus* (63%), aerobic gram-negative bacilli (16%), aerobic streptococci (9%), *Staphylococcus epidermidis* (3%), anaerobes (2%), others (1%), and unknown (6%) [7]. Extremely rare, has SEA been caused by *Streptococcus agalactiae* [1–4]. We believe, however, that this is the first case in English-language literature in which *S. agalactiae* caused both lumbar SEA and facet joint septic arthritis, then spread to the iliopsoas and paraspinal muscles. It was detected in the patient’s urine, blood and tissues.

Group B Streptococcus (GBS), was initially considered a neonatal pathogen [8], but recently its appearance in neonates has decreased [9]. In contrast, its diagnosis and mortality rate in people aged ≥65 years has increased compared with that of adolescents and adults 15–64 years of age [9, 10]. GBS septic arthritis generally affects only a single joint, most often the knee, hip, or shoulder [11].

Although septic arthritis generally affects the larger peripheral joints, it has also been reported occasionally in facet joints, with the lumbar spine being the mostly frequently involved (86–97%) [12]. The commonest mode of spread is via the hematogenous route from an infection elsewhere in the body (72%) [12]. Other such infections have arisen iatrogenic from procedures e.g. corticosteroid injection [13], epidural injection, acupuncture [14], direct introduction via surgery and indirectly via penetrating trauma (e.g. a human or animal bite or nail puncture), or after trauma to a joint without an obvious break in the skin [15]. Facet joint infections can spread, with 4–20% of them leading to pyogenic spinal infection [12] and then infection of the paraspinal muscle and epidural space. They may also cause an intramuscular lesion of the psoas muscle [16]. Unlike septic arthritis due to *Staphylococcal aureus*, *S. agalactiae* rarely involves facet joints. A few cases have been reported in which the mode of spread was infective endocarditis [3]. In our case the mode of spread was hematogenous.

The three most common presenting symptoms of SEA are back pain, fever and neurological deficit [17]. These symptoms however do not always occur together [5], thus contributing to the delayed diagnosis which reportedly occurs in 50–75% of cases [18]. Hence, clinicians must maintain a high level of suspicion for spinal epidural abscess so as to diagnose and treat it to avoid development of irreversible deficits [5].

In our patient, lumbar SEA and facet joint septic arthritis due to *S. agalactiae* was diagnosed by detecting elevated levels of inflammatory markers in blood: high erythrocyte sedimentation rate, elevated CRP level, and leukocytosis. Blood and tissue cultures served an important role in identifying the causative organism. Like in the present case, MRI has shown to be an important modality in diagnosing spine infection, with one of its major role being to look for the spread of infection in the epidural space and spinal canal with any effects on the cord and cauda equina nerve roots [19].

Spinal epidural space anatomy offers little resistance to the longitudinal spread of the infection [7], indicating the need for an urgent management. Facet joint arthritis is mostly treated conservatively by antibiotic administration, initially intravenously and then orally [20]. For an abscess in paraspinal muscle, percutaneous drainage of the intramuscular abscess has a high rate of success [16]. Abscesses in the epidural space have been subjected to both conservative [2, 21] and operative [22] therapy. Patients with complications such as cauda equina syndrome should urgently undergo surgery: opening and draining the facet joints and possibly decompressive laminectomy [20]. Doita et al. [23] reported that they treated facet arthritis surgically because intravenous antibiotic therapy was ineffective. Our patient with septic arthritis of the lumbar facet joints was treated operatively despite diminishing signs of inflammation. We considered that even though the culprit bacteria was identified and a sensitive antibiotic prescribed, the patient’s neurologic symptoms could be exacerbated by spread of the abscess. In such cases, surgical resection should be performed immediately. Also, routine administration of a suitable GBS vaccine to adults ≥65 years of age can help reduce the mortality and morbidity due to invasive GBS infections [24].

In conclusion considering the rarity of this disease, clinicians’ suspicion should rise for any patient who presents with back pain, fever, and spine tenderness. Evaluation of urine and blood specimen, and MRI are sufficient modalities to reach the diagnosis. Early surgical exploration and broad spectrum-antibiotics are essential to attain good recovery and a favourable outcome.

## Abbreviations

CRP: C-reactive protein levels
ESR: Erythrocyte sedimentation rate
MMT: Manual muscle test
MRI: Magnetic resonance imaging
SEA: Spine epidural abscess
UTI: Urinary tract infection
